# Comparing the Clinical and Economic Outcomes Associated with Adjuvanted versus High-Dose Trivalent Influenza Vaccine among Adults Aged ≥ 65 Years in the US during the 2019–20 Influenza Season—A Retrospective Cohort Analysis

**DOI:** 10.3390/vaccines9101146

**Published:** 2021-10-08

**Authors:** Myron J. Levin, Victoria Divino, Drishti Shah, Mitch DeKoven, Joaquin Mould-Quevedo, Stephen I. Pelton, Maarten J. Postma

**Affiliations:** 1 Departments of Pediatrics and Medicine, Anschutz Medical Campus, University of Colorado, Aurora, CO 80045, USA; Myron.Levin@cuanschutz.edu; 2 Real World Solutions, IQVIA, Falls Church, VA 22042, USA; drishti.shah@iqvia.com (D.S.); mitch.dekoven@iqvia.com (M.D.); 3 Global Pricing & Health Economics, Seqirus USA Inc., Summit, NJ 07901, USA; Joaquin.Mould-Quevedo@seqirus.com; 4 Department of Pediatrics, Boston University Schools of Medicine, Boston, MA 02118, USA; spelton@bu.edu; 5 Division of Pediatric Infectious Diseases, Maxwell Finland Laboratory, Boston Medical Center, Boston, MA 02118, USA; 6 Department of PharmacoTherapy, Epidemiology & Economics (PTE2), Groningen Research Institute of Pharmacy, University of Groningen, 9713 AV Groningen, The Netherlands; m.j.postma@rug.nl; 7 Department of Health Sciences, University Medical Center Groningen, University of Groningen, 9713 AV Groningen, The Netherlands; 8 Department of Economics, Econometrics & Finance, Faculty of Economics & Business, University of Groningen, 9749 AE Groningen, The Netherlands

**Keywords:** influenza, older adults, adjuvanted influenza vaccine, relative vaccine effectiveness, retrospective studies, economic outcomes

## Abstract

The burden of influenza is disproportionally higher among older adults. We evaluated the relative vaccine effectiveness (rVE) of adjuvanted trivalent (aIIV3) compared to high-dose trivalent influenza vaccine (HD-IIV3e) against influenza and cardio-respiratory disease (CRD)-related hospitalizations/ER visits among adults ≥65 years during the 2019–2020 influenza season. Economic outcomes were also compared. A retrospective cohort analysis was conducted using prescription, professional fee claims, and hospital data. Inverse probability of treatment weighting (IPTW) was used to adjust for confounding. IPTW-adjusted Poisson regression was used to evaluate the adjusted rVE of aIIV3 versus HD-IIV3e. All-cause and influenza-related healthcare resource utilization (HCRU) and costs were examined post-IPTW. Recycled predictions from generalized linear models were used to estimate adjusted costs. Adjusted analysis showed that aIIV3 (*n* = 798,987) was similarly effective compared to HD-IIV3e (*n* = 1,655,979) in preventing influenza-related hospitalizations/ER visits (rVE 3.1%; 95% CI: −2.8%; 8.6%), hospitalizations due to any cause (−0.7%; 95% CI: −1.6%; 0.3%), and any CRD-related hospitalization/ER visit (0.9%; 95% CI: 0.01%; 1.7%). Adjusted HCRU and annualized costs were also statistically insignificant between the two cohorts. The adjusted clinical and economic outcomes evaluated in this study were comparable between aIIV3 and HD-IIV3e during the 2019–2020 influenza season.

## 1. Introduction

Older adults (aged ≥ 65 years) are at higher risk of infection with seasonal influenza and subsequent complications compared to younger adults [1,2]. Influenza-related morbidity and mortality is highest among older adults due to higher comorbidity burden, immunosenescence and reduced vaccine effectiveness, with the risk of death nearly doubled in those aged ≥75 years compared with those aged 65–74 years [1,3]. During the 2019–2020 influenza season, older adults accounted for 45% of estimated influenza-related hospitalizations and 59% of estimated influenza-related deaths in the United States (US) [4].

Prevention by vaccination, especially among older adults, is the most practical, cost-effective, and minimally disruptive method of preventing influenza [1]. During the 2019–2020 influenza season in the US, two egg-based vaccines, an adjuvanted trivalent influenza vaccine (aIIV3; Fluad^®^, Seqirus, Summit, NJ, USA) and high-dose trivalent influenza vaccine (HD-IIV3e; Fluzone High-Dose^®^, Sanofi Pasteur, Bridgewater, NJ, USA) were available and approved for use among older adults [5]. According to the Centers for Disease Control and Prevention (CDC), there is no preferential recommendation for any one vaccine approved for use among older adults [3]. aIIV3 and HD-IIV3e present opportunities to reduce influenza and related burden among older adults compared to standard influenza vaccines [1,6]. Higher efficacy, effectiveness and immunogenicity of enhanced vaccines compared to standard dose vaccines have been demonstrated by several studies [1,6,7,8,9]. Details of the two enhanced vaccines are described in another study by the authors [10].

In the absence of randomized controlled trials comparing the efficacy of aIIV3 and HD-IIV3e, real-world data has been used to evaluate the relative vaccine effectiveness (rVE) of aIIV3 and HD-IIV3e [10,11,12,13,14,15]. Two studies from the 2018–2019 influenza season found comparable clinical outcomes between aIIV3 and HD-IIV3e [10,14]. The first study conducted among a Medicare fee-for-service (FFS) population found that aIIV3 was associated with a statistically insignificant rVE against influenza-related hospital encounters compared to HD-IIV3e [14]. The second study also found rVE to be comparable in the prevention of influenza-related hospitalization/emergency room (ER) visits. Additionally, aIIV3 was slightly more effective in preventing any cardio-respiratory (CRD)-related hospitalization/ER visit [10]. Healthcare costs post-vaccination were also evaluated in the second study and found to be comparable [10]. Given that influenza seasons vary each year in terms of timing, duration, activity, and clinical severity due to changes in antigenic composition of the influenza virus [1], it is important to update estimates for rVE and economic burden each year. Unlike the prior seasons, the 2019–2020, influenza activity in the US began to increase in November and was constantly high throughout January and February [4]. Activity began to decline in March. The 2019–2020 influenza season was described as having moderate severity and was predominantly H1N1. Any evaluation of the 2019–2020 influenza season must take into account the impact of the coronavirus disease 2019 (COVID-2019) [4].

Only one retrospective study has so far been published that evaluated rVE of aIIV3 and HD-IIV3e for the 2019–2020 influenza season [12]. This study was conducted among Medicare FFS beneficiaries and, in line with prior studies, no statistically significant difference was observed in influenza-related hospital encounters between recipients of aIIV3 and HD-IIV3e. Because influenza is associated with an additional burden of non-respiratory complications, which include cardiovascular events, cerebrovascular events, exacerbations of chronic underlying conditions, and functional decline, it is important to evaluate these clinical outcomes, as well as the overall economic burden of influenza among older adults [16]. The objective of this study was to evaluate the rVE of aIIV3 compared to HD-IIV3e against clinical outcomes related to influenza-related hospitalizations/ER visits and CRD-related hospitalizations/ER visits during the 2019–2020 influenza season among older adults in the US using nationally representative data. This study also examined economic outcomes, including healthcare resource utilization (HCRU) and costs (all-cause and influenza-related) between aIIV3 and HD-IIV3e patients.

## 2. Methods

### 2.1. Study Design

This study used a retrospective observational cohort design. It was conducted among patients aged ≥65 years vaccinated with aIIV3 or HD-IIV3e during the 2019–2020 influenza season in the US using de-identified data from IQVIA’s New Data Warehouse: Professional Fee Claims (Dx), Prescription Claims (Rx), and Hospital Charge Data Master (CDM) databases. Ethics approval was not required to use these de-identified secondary data sources.

### 2.2. Data Sources

The analytical dataset was constructed from the deterministically linked Dx, Rx, and CDM databases. The datasets were linked using actual patient information (e.g., gender, date of birth, zip code, etc.) to assign a unique patient ID [17]. Dx data includes approximately 1 billion professional fee claims per year, representing over 870,000 practitioners per month and 70–75% of physician activity in the US. Rx data represents approximately 85% of all pharmacies in the US. It includes more than 1.6 billion retail or mail-order prescription claims. CDM includes records from over 450 hospitals, covering 7 million inpatient stays and 60 million outpatient visits per year. It includes records from hospital charge data master files, the service order records drawn from hospital operational files and other reference sources. The databases are compliant with the Health Insurance Portability and Accountability Act (HIPAA) to protect patient’s privacy. These data sources include all payers (including traditional Medicare FFS) and are representative of adults ≥65 years old.

### 2.3. Study Population

The starting population of interest was patients aged ≥65 years with at least 1 medical or pharmacy claim for aIIV3 or HD-IIV3e in Rx or Dx during the vaccination window, 4 August 2019 to 31 January 2020. The 2019–2020 influenza season was defined from 4 August 2019 through 7 March 2020. The study period began 4 February 2019, allowing for a 6-month baseline or pre-index period (to assess study eligibility criteria and measure patient baseline characteristics) and ended 7 March 2020. While the actual 2019–2020 influenza season extended beyond 7 March 2020, for the purpose of this study we ended our study period on 7 March 2020 to minimize any outcome misclassification that might be caused by the COVID-19 pandemic [18].

**Figure 1:**
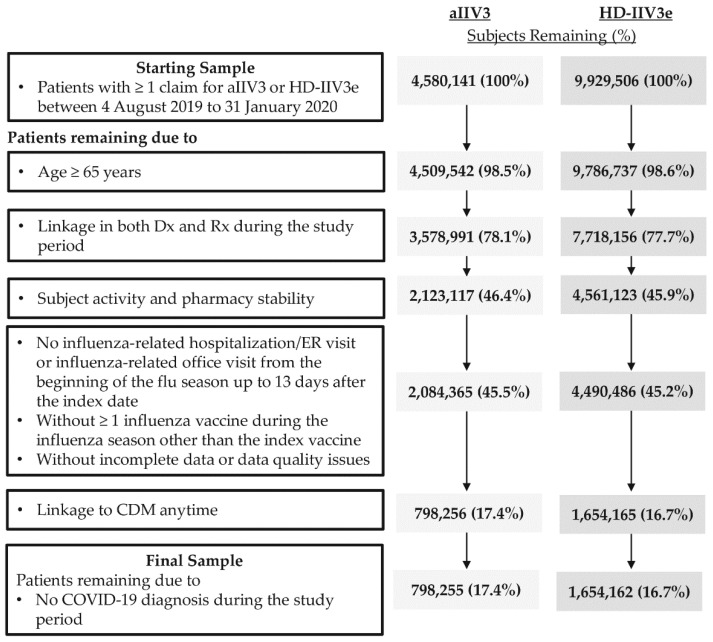
Patient Selection. Abbreviations. aIIV3 = adjuvanted trivalent influenza vaccine; CDM = Hospital Charge Data Master; COVID-19 = coronavirus disease 2019; Dx = Professional fee claims; ER = Emergency room; HD-IIV3e = high-dose trivalent influenza vaccine; Rx = Prescription claims.

The date of the first claim for aIIV3 or HD-IIV3e in the vaccination window was termed the “index date” and determined the vaccine cohort. The two cohorts were mutually exclusive. The following additional inclusion criteria were required: (1) Linkage in both Dx and Rx during the study period; (2) Patient activity, defined as ≥1 office visit (in Dx) and ≥1 prescription (in Rx) the 6 months prior to the 6-month pre-index period, as well as in the 6 months following the end of the influenza season (8 March 2020–7 September 2020); (3) Pharmacy stability in the 6-month pre-index period through the end of the influenza season, defined as consistent reporting of data from the pharmacy most frequently visited by the patient from the start of the 6-month pre-index period through the end of the influenza season. In addition, patients that met any of the following exclusion criteria were removed from the study: (1) Those with an influenza-related hospitalization or ER visit or an influenza-related office visit (subsequently defined in Section 2.4) between the index date up to 13 days after the index date; (2) Those who received any other influenza vaccine during the 2019–2020 influenza season other than index influenza vaccine; (3) Patients with incomplete data or data quality issues (missing gender, region or payer type); (4) Patients without linkage to CDM at any time; and (5) Patients with ≥1 claim with a COVID-19 diagnosis (ICD-10 code U07.1) [19] during the study period. The study eligibility criteria were generally consistent with prior studies [10,15]. Details of step by step application of study eligibility criteria are presented in Figure 1.

### 2.4. Study Measures

#### 2.4.1. Patient Characteristics

Baseline demographic and clinical characteristics can be found in Table 1 and Table 2. These included demographic characteristics measured as of the index date, such as age, gender, US Department of Health & Human Services (DHHS) region, and payer type. Clinical characteristics including month of influenza vaccination as well as Charlson Comorbidity Index (CCI; Dartmouth-Manitoba adaptation), comorbidities of interest [13,14], indicators of frail health status [13,14], indicators of health seeking behavior [12], and pre-index all-cause costs which were measured over the baseline period. A cost:charge ratio (CCR) was applied to the charges in Dx and CDM, because only charges are available in these databases, using hospital outpatient prospective payment system (OPPS) CCR files from the Centers for Medicare & Medicaid Services (CMS) and Inpatient CCR files from the Healthcare Cost and Utilization Project (HCUP), respectively [20,21].

#### 2.4.2. Outcome Assessment

Study outcomes were assessed over the variable post-index or follow-up period which began 14 days after the index date (in order to allow for the development of vaccine-specific immunity) through the end of the influenza season (7 March 2020). The assessment of any outcomes of interest related to hospitalizations that had an admission date on or before 7 March 2020, but a discharge date past 7 March 2020, considered the entire hospitalization for the assessment.

#### 2.4.3. Clinical Outcomes

Clinical outcomes included number and rates (events per 1000 vaccinated patients) of the following events: influenza-related hospitalization/ER visits, CRD-related hospitalization/ER visits (comprising of events related to any CRD event as well as specific pneumonia, asthma/COPD/bronchial, coronary artery [including myocardial infarction (MI)], congestive heart failure, or cerebrovascular [including stroke] events) [15], and all-cause hospitalizations. The methodology and definitions are in line with studies comparing aIIV3 vs. HD-IIV3e in previous influenza seasons [10,15]. Influenza-related hospitalizations and ER visits were identified based on a diagnosis code for influenza (ICD-9 487.x, 488.x, ICD-10 J09.x, J10.x, J11.x) [22] in any position [13,14]. Any CRD hospitalization/ER visit was defined based on a CRD-related diagnosis code (ICD-9 390-519, ICD-10 Ixx-Jxx) in any position. Select CRD hospitalizations/ER visits were also identified based on a diagnosis code in any position for the event of interest.

A control outcome of hospitalizations related to urinary tract infection (UTI) was also evaluated [11]. UTI-related hospitalization was defined based on a diagnosis code for UTI in any position. Influenza vaccine is not expected to prevent UTI; therefore, reporting a control outcome can be used to demonstrate a similar treatment effect across the two vaccines. For each outcome of interest, the first occurring event was identified at the subject level. An individual could contribute an event for more than one outcome.

#### 2.4.4. Economic Outcomes

Economic outcomes of interest included all-cause HCRU and costs for the following mutually exclusive healthcare categories: outpatient pharmacy, inpatient hospitalizations, ER visits and outpatient medical. Influenza-related HCRU and associated costs were assessed specific to the previously defined influenza-related hospitalizations/ER visits, as well as influenza-related office visits (office visits with an influenza diagnosis code in any position) and influenza-related pharmacy (influenza-related antiviral medications). Utilization and costs were calculated on a per patient basis, averaged across the cohort. Costs were annualized and reported in 2019 USD.

### 2.5. Statistical Analyses

Descriptive statistics (frequency and percentage for categorical measures and mean, standard deviation [SD], median for continuous measures) were reported for each study cohort. Standardized mean differences (SMD, calculated as difference in means or proportions of a variable divided by the pooled SD) were reported to evaluate the difference in baseline patient characteristics between the vaccine cohorts. SMD (absolute) of ≥0.10 was considered to be statistically meaningful [23].

In order to adjust for imbalances in measured confounders and treatment selection bias, inverse probability of treatment weighting (IPTW) was implemented. Our statistical approach has been described in further detail in prior publications [10,15]. IPTW is used to create a weighted sample (or “pseudo-population”) in which the distribution of measured covariates is the same between treated cohorts [23]. IPTW weights were derived from propensity scores, which were calculated using a logistic regression model with vaccine cohort as the dependent variable. Baseline variables with SMD ≥ 0.10 prior to IPTW and other clinically relevant variables (age group, gender, payer type, DHHS region, month of influenza vaccine, CCI score [categorical], any frailty indicator [yes/no] and pre-index hospitalization [yes/no]) were included as independent variables. The current study used stabilized weights in order to reduce type I error [24,25]. Due to the potential bias of outliers, weight values greater than ten were truncated to ten [12]. Clinical and economic outcomes were evaluated following IPTW.

For clinical outcomes, adjusted rate ratios (RR) and 95% CIs for aIIV3 compared to HD-IIV3e were estimated using Poisson regression. Poisson regression models allowed for a robust regression adjustment and further reduced any residual confounding. Adjusted rVE was calculated as ([1-RR] × 100%). Baseline characteristics were well balanced following IPTW; therefore, the univariate Poisson regression models only included IPTW weight.

For economic outcomes, pairwise comparisons of HCRU and costs between aIIV3 and HD-IIV3e cohorts post-IPTW were conducted using weighted chi-square tests for categorical variables and weighted *t*-tests (mean) for continuous variables. Predicted annualized mean costs were generated for: (1) all-cause total healthcare costs and (2) influenza-related total costs, comprising (3) hospitalization costs, (4) ER costs, (5) office visit costs and (6) pharmacy costs related to influenza. Generalized linear regression (GLM) models were developed post-IPTW to estimate predicted costs using a counterfactual recycled predictions approach [26,27]. For annualized all-cause total healthcare costs, a weighted GLM with log link and gamma distribution was developed. Because influenza-related events were less frequent, two-part weighted GLM models were developed for the remaining outcomes. The first model had a binomial distribution and logit link to estimate odds of having a non-zero cost for the outcome of interest (i.e., of having the outcome). The second model used a gamma distribution and log link to estimate the cost of the outcome of interest, among patients with the outcome of interest. Adjustment for outliers was made by capping cost at the 99th percentile for all patients (all-cause total cost) or among those with at least 1 such outcome (influenza-related costs). Predicted recycled means were obtained from the parameter estimates of GLMs and their 95% CIs (2.5th percentile and 97.5th percentile assuming a non-normal distribution) were derived through bootstrapping (500 replications). Unlike GLM, which uses a reference-case scenario, recycled predictions use an average-case scenario [27]. Similar to Poisson regression models developed for the clinical outcomes, weighted GLMs included IPTW weight only.

All analyses were based on observed data without projection. SAS^®^ Release 9.4 (SAS Institute Inc., Cary, NC, USA) was utilized for the analyses.

### 2.6. Sub-Group and Sensitivity Analysis

Sub-group and sensitivity analyses were conducted for select clinical outcomes of interest: hospitalizations/ER visits related to influenza and CRD.

Sub-group analyses were conducted for 3 age groups (65–74 years, 75–84 years, and ≥85 years), with separate IPTW.

Three sensitivity analyses including use of varying measurement periods and advanced statistical methodologies were conducted in this study for the select clinical outcomes of interest. The first sensitivity analysis restricted the observation period to the high influenza activity period (HIAP) with assessment from [(index date + 14) or 8 December 2019], whichever occurred later, to 7 March 2020 (Week 50 to 10). The HIAP was determined through a Moving Epidemic Method (MEM) algorithm to establish epidemic thresholds for the influenza season [28]. Additional details on this algorithm have been described in a prior study [10].

The second sensitivity analysis was conducted for a shortened influenza period with measurement period starting from 4 August 2019 but ending 15 February 2020 (end of Week 7). This shortened influenza period was evaluated to help assess the potential impact of earlier than expected community transmission of COVID-19.

Following methodology from recent studies, the third sensitivity analysis used a doubly robust analysis in order to test the robustness of the findings from the main analysis [12,14,29]. The outcome regression model of the doubly robust analysis included the independent variables used in the logistic regression model to derive weights for IPTW (described above) as well as the IPTW weight. Doubly robust adjustment is used to account for any residual confounding from measured covariates [30].

## 3. Results

### 3.1. Study Sample

The starting sample comprised 4,580,141 aIIV3 and 9,929,506 HD-IIV3e recipients during the vaccination window of the 2019–2020 influenza season. aIIV3 and HD-IIV3e recipients were most often excluded due to not meeting the requirements for patient activity and pharmacy stability. The final unadjusted sample comprised 798,255 aIIV3 and 1,654,162 HD-IIV3e recipients (Figure 1), with a median follow-up period of 5 months for both cohorts.

### 3.2. Patient Characteristics

aIIV3 and HD-IIV3e patients had a mean age of 75.0 years. A few baseline characteristics were imbalanced with SMD (absolute) ≥0.1 prior to IPTW. A higher proportion of aIIV3 patients were located in the South (52.0%) compared to HD-IIV3e (45.3%) and a lower proportion had third-party insurance (23.7% and 29.5%, respectively). The proportion of aIIV3 patients vaccinated in the month of August was also higher (10.7% and 1.5%, respectively). Post-IPTW, aIIV3 (*n* = 798,987) and HD-IIV3e (*n* = 1,655,979) recipients were well-balanced across all measured baseline characteristics. Subject baseline demographic and clinical characteristics pre- and post-IPTW are presented in Table 1 and Table 2.

### 3.3. Clinical Outcomes

**Figure 2:**
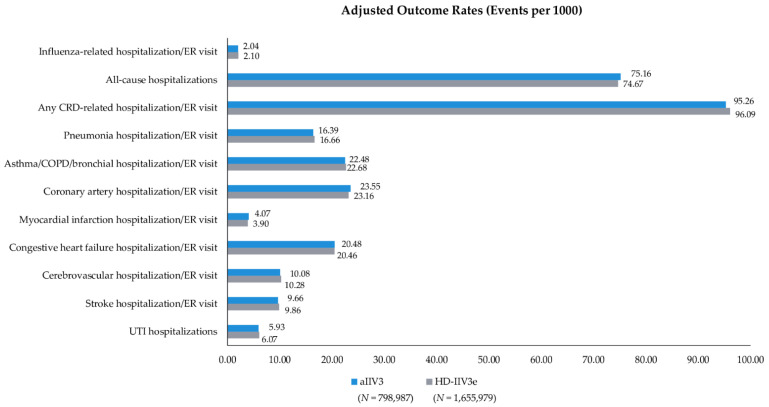
Adjusted Outcome Rates—Post-IPTW—aIIV3 vs. HD-IIV3e. Rate = events per 1000 vaccinated patients; aIIV3 = adjuvanted trivalent influenza vaccine; CRD = cardio-respiratory disease; ER = emergency room; HD-IIV3e = high-dose trivalent influenza vaccine; UTI = urinary tract infection.

**Figure 3:**
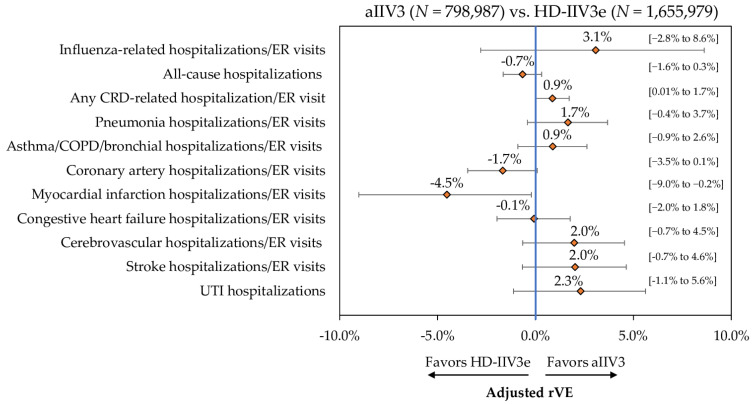
Adjusted rVE (95% CI)—Post-IPTW and Poisson Regression—aIIV3 vs. HD-IIV3e from Main Analysis. aIIV3 = adjuvanted trivalent influenza vaccine; CRD = cardio-respiratory disease; ER = emergency room; HD-IIV3e = high-dose trivalent influenza vaccine; rVE = relative vaccine effectiveness; UTI = urinary tract infection.

Event rates post-IPTW can be found in Figure 2 and IPTW and Poisson regression adjusted rVEs are presented in Figure 3 and Table S1. Following IPTW and Poisson regression adjustment, aIIV3 was statistically comparable to HD-IIV3e for almost all clinical outcomes of interest. aIIV3 was comparable to HD-IIV3e in preventing hospitalizations/ER visits related to influenza (rVE = 3.1%; 95% CI: −2.8%; 8.6%) and CRD (rVE = 0.9%; 95% CI: 0.01%; 1.7%), as well as in preventing all-cause hospitalizations (rVE = −0.7%; 95% CI: −1.6%; 0.3%). aIIV3 was also comparable to HD-IIV3e in preventing hospitalizations/ER visits related to pneumonia, asthma/COPD/bronchial, coronary artery disease, congestive heart failure, and cerebrovascular events (including stroke). However, aIIV3 was slightly less effective against MI (rVE = −4.5%; 95% CI: −9.0%; −0.2%). No statistical difference (i.e., treatment effect) was observed in the control outcome of UTI hospitalizations between the two vaccine cohorts.

### 3.4. Economic Outcomes

Proportion of patients with ≥1 influenza-related hospitalization (0.09% vs. 0.09%; *p* = 0.5800) and ER visit (0.15% vs. 0.15%; *p* = 0.1626) was similar across aIIV3 and HD-IIV3e cohorts. Similarly, proportion of patients with ≥1 influenza-related office visit was similar (0.41% vs. 0.42%; *p* = 0.1944) (Figure S1). Post-IPTW influenza-related annualized costs are presented in Figure S2 and all-cause HCRU and annualized costs are presented in Table S2.

Following GLM adjustment, aIIV3 and HD-IIV3e were associated with comparable predicted mean annualized costs for the evaluated economic outcomes: all-cause total healthcare costs, influenza-related total healthcare costs, and influenza-related component costs for hospitalizations ER visits, office visits, and pharmacy (Table 3). For example, annualized all-cause and influenza-related total costs were statistically comparable between aIIV3 and HD-IIV3e (USD 13,196 vs. USD 13,221 and USD 21.64 vs. USD 21.92, respectively; both *p* > 0.05).

### 3.5. Sub-Group and Sensitivity Analyses

**Figure 4:**
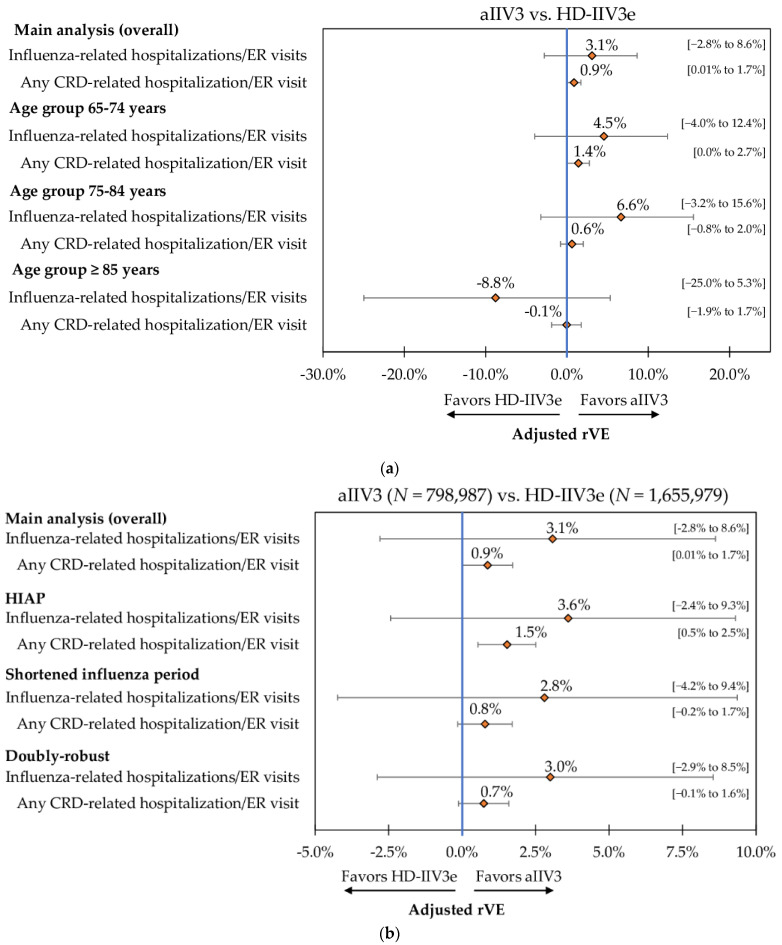
(**a**) Adjusted rVE (95% CI)—Post-IPTW and Poisson Regression—aIIV3 vs. HD-IIV3e from Age Sub-group Analysis. (**b**) Adjusted rVE (95% CI)—Post-IPTW and Poisson Regression—aIIV3 vs. HD-IIV3e from Sensitivity Analysis. aIIV3 = adjuvanted trivalent influenza vaccine; CRD = cardio-respiratory disease; ER = emergency room; HD-IIV3e = high-dose trivalent influenza vaccine; rVE = relative vaccine effectiveness; UTI = urinary tract infection.

Results for the select clinical outcomes of interest from sub-group and sensitivity analyses were generally consistent with the main analysis. When analyzed by age sub-group, aIIV3 was comparable to HD-IIV3e against hospitalizations/ER visits related to influenza and CRD (Figure 4A and Table S1).

Results from the sensitivity analyses were also generally consistent with the main overall analysis. After IPTW adjustment and Poisson regression, aIIV3 was comparable to HD-IIV3e against influenza-related hospitalizations/ER visits across all three sensitivity analyses. During the HIAP, aIIV3 was more effective against hospitalizations/ER visits related to CRD (rVE = 1.5%; 95% CI: 0.5%; 2.5%), but with a relatively small effect. However, during the shortened influenza period and in the doubly robust analysis, the two vaccine cohorts had comparable effectiveness against this outcome (Figure 4B and Table S1).

## 4. Discussion

As vaccination is the primary preventative tool against severe influenza disease, especially among the elderly, it is critical to understand the relative effectiveness of vaccines approved for this high-risk population. This real-world analysis compared the rVE of aIIV3 to HD-IIV3e among a representative population of older adults (aged ≥ 65 years) during the 2019–2020 influenza season using claims and hospital data in the US. This study employing robust methodology was conducted among >2.4 million enhanced influenza vaccine recipients (aIIV3 and HD-IIV3e) aged ≥65 years during the 2019–2020 influenza season and found comparable effectiveness between the two vaccines against a comprehensive set of clinical outcomes. Following the pairwise IPTW adjustment, aIIV3 and HD-IIV3e cohorts were well-balanced across the measured baseline characteristics. Following IPTW-weighted Poisson regression, aIIV3 was associated with comparable rVE against hospitalizations/ER visits related to influenza and CRD, as well as against all-cause hospitalizations. HCRU and costs, both all-cause and influenza-related, were also comparable between the vaccine cohorts.

Overall, the observed rates (per 1000 vaccinated patients) of influenza-related hospitalizations ER/visits among the aIIV3 and HD-IIV3e cohorts (2.04 and 2.10) were lower in the current study (2019–2020 influenza season) compared to prior influenza seasons. For example, the authors’ analysis of the 2017–2018 influenza season (a high severity influenza season) found adjusted rates (per 1000) of 5.37 and 5.62 among aIIV3 and HD-IIV3e recipients, respectively [15]. The current lower rates may be related to the shortened study period implemented in order to avoid potential outcome misclassification due to the COVID-19 pandemic as any assessment of influenza-related burden during 2019–2020 influenza season must consider the presence and impact of the COVID-19 pandemic. It is also notable that there was little to no significant circulation of A(H3N2) during the 2019–2020 influenza season, which is often a major contributor to influenza-related hospitalizations and mortality among elderly [12,31].

The current study used robust IPTW adjusted regression analysis for handling treatment selection bias for outcomes assessment. The approach of using IPTW which is one of the more robust techniques for adjusting covariate imbalance between cohorts [32] is in line with several published studies that have compared influenza vaccine effectiveness [10,12,14]. Additionally, for economic outcomes assessment, rather than simply comparing costs for a reference-case scenario, counterfactual recycled prediction technique was used to compute robust estimates of incremental cost between the two cohorts. This method also helps avoid the problem of covariate imbalance in the two cohorts by creating an identical covariate structure, i.e., similar individual patient characteristics (counterfactual scenarios) in both aIIV3 and HD-IIV3e cohorts [26,27]. To that end, following such robust analyses, the two enhanced vaccines (aIIV3 and HD-IIV3e) were comparable across the clinical and economic outcomes evaluated in the current study. We found that aIIV3 was statistically insignificant to HD-IIV3e in preventing influenza-related hospitalizations/ER visits and all-cause hospitalizations. To our knowledge, one other real-world retrospective study has evaluated rVE of aIIV3 and HD-IIV3e during the 2019–2020 season among Medicare FFS beneficiaries. That study may not be nationally representative of individuals aged ≥65 years [12], and furthermore, the study outcomes were limited to influenza-related hospital encounters. However, HD-IIV3e was found to have similar effectiveness compared to aIIV3 against preventing influenza-related hospital encounters (−1.6%, 95% CI: −4.8%; 1.6%) [12], in line with the current study findings.

As the effect of influenza extends well beyond respiratory disease [16], it is important to assess the comparative effectiveness of influenza vaccines against other relevant clinical outcomes. In particular, cardiovascular events have been identified as the most common chronic condition among hospitalized patients with influenza [33]. Influenza vaccination is also associated with a reduction in the incidence of adverse cardiac events among those with co-existing cardiovascular diseases [33,34]. In addition to influenza-related hospitalization outcomes, the current study also examined rVE against hospitalizations/ER visits related to CRD as well as several specific CRD events of interest. Similar effectiveness between aIIV3 and HD-IIV3e against influenza- and CRD-related hospitalizations/ER visits as seen from the current study and from other studies conducted by the authors from prior influenza seasons [10,15] suggests no preferential advantage of one enhanced vaccine over another. However, additional studies will be needed to understand if this finding will hold true for additional influenza clades during future influenza seasons.

In line with the results from the main analysis, our findings were also robust in the age sub-group and sensitivity analyses. As the risk of morbidity and mortality due to influenza increases with age among older adults [1], it is important to assess the effectiveness of vaccines by age sub-groups. We observed that the rVE against influenza-related hospitalizations/ER visits and any CRD hospitalization/ER visit was similar between aIIV3 and HD-IIV3e across the three age-groups (65–74, 75–84 and ≥85 years), as well as across the HIAP and shortened flu season and in the doubly robust analysis, with one main exception. aIIV3 was more effective than HD-IIV3e against any CRD-related hospitalization/ER visit during the HIAP (rVE = 1.5%; 95% CI: 0.5%; 2.5%).

In addition to health impact, seasonal influenza also has a significant economic impact. Older adults contribute to the majority of influenza-related economic burden in the US [35]. Economic data are an essential part for effective decision-making by policy makers. Therefore, while clinical effectiveness was found to be similar between aIIV3 and HD-IIV3e during the 2019–2020 influenza season, it is also important to assess whether this translates to similar economic outcomes which can further help to inform policy decisions. This is the first real-world study to compare economic outcomes between aIIV3 and HD-IIV3e during the 2019–2020 influenza season. In line with clinical outcomes, the current study also showed similar economic outcomes in terms of all-cause and influenza-related HCRU and costs between the two vaccine cohorts. Annualized predicted total all-cause and influenza-related costs were statistically insignificant between aIIV3 and HD-IIV3e (USD 13,196 vs. 13,221; *p* = 0.2720 and USD 21.64 vs. 21.92; *p* = 0.4000). The findings from this study are also consistent with prior studies from the 2017–2018 and 2018–2019 influenza seasons [10,15]. More real-world studies are needed to further explore potential cost-savings associated with these vaccines using different data sources and study populations.

Our study findings need to be interpreted in light of limitations. First, due to the retrospective observational nature of the study design, all study findings are associative, and no causal inferences can be made. Second, the methods used to adjust for treatment selection bias are based on measurable factors only and does not take into account unmeasured factors such as race, socio-economic status, access to care, etc. which may impact vaccine utilization and potentially the study outcomes of interest. With that said, highly balanced cohorts were achieved after IPTW adjustment with respect to measured variables. Additionally, after IPTW adjustment, no significant difference was observed in UTI hospitalizations (control outcome) between the two vaccine cohorts suggesting that that the two cohorts were comparable in characteristics. Additionally, indicators of health seeking behaviors were also well-balanced between the two cohorts. Third, there are important limitations related to the use of open-source claims databases that should be considered. Only healthcare activity/consumption from pharmacies/offices/hospitals that contribute to the databases are captured. The requirement of linkage to CDM (any time during the available CDM data) may bias the study sample towards a more severe population due to activity in a hospital contributing to the CDM data. However, since this requirement was applied to both the vaccine cohorts, we do not anticipate differential impact across any one cohort. Fourth, due to the use of healthcare claims which lack clinical detail, there is a potential for miscoding or misclassification. In particular, lab or test results were unavailable to confirm influenza infection. Additionally, fifth, the current study examined only direct healthcare costs. Other indirect and societal costs (e.g., costs associated with loss of productivity, caregiving, travel time, etc.) could not be captured from the study datasets, thereby limiting a comprehensive total cost assessment. It should also be noted that because the outcomes assessment period began 14 days after the index date, the price of the two vaccines was not included in the healthcare cost assessment. However, the average sales price (ASP) of aIIV3 and HD-IIV3e is similar [36].

## 5. Conclusions

aIIV3 and HD-IIV3e were comparable in the prevention of influenza-related hospitalizations/ER visits and hospitalizations/ER visits related to CRD during the 2019–2020 influenza season. The data were robust across the age sub-group analysis and the three sensitivity analyses and consistent with the main analysis. HCRU and costs, both all-cause and influenza-related, were also comparable between cohorts suggesting the vaccines provided similar benefit across the measured outcomes of interest. The analyses conducted by the authors using real-world data over this and other influenza seasons suggest that both enhanced vaccines have comparable clinical and economic benefits and have similar effectiveness in preventing complications due to influenza among older adults. This is valuable information for healthcare policy makers regarding influenza control.

## Figures and Tables

**Table 1:** Baseline Demographic Characteristics—Pre- and Post-IPTW.

	Pre-IPTW			Post-IPTW		
Vaccine Cohort Characteristics	aIIV3 *n* = 798,255	HD-IIV3e *n* = 1,654,162	SMD ^1^	aIIV3 *n* = 798,987	HD-IIV3e *n* = 1,655,979	SMD ^1^
**Mean age**	75.0	75.0	−0.01	75.0	75.0	0.00
SD	6.2	6.3		6.2	6.2	
Median	74	74		74	74	
**Age group (%)**						
65–74 years	50.5%	51.0%	0.01	50.9%	50.9%	0.00
75–84 years	34.8%	34.2%	−0.01	34.3%	34.4%	0.00
≥85 years	14.6%	14.8%	0.01	14.7%	14.7%	0.00
Female (%)	58.9%	59.4%	0.01	59.2%	59.3%	0.00
**Geographic region (%)**						
Northeast	15.6%	18.1%	0.07	17.7%	16.9%	−0.02
Midwest	16.7%	18.0%	0.03	17.5%	17.6%	0.00
South	52.0%	45.3%	**−0.13**	47.0%	47.9%	0.02
West	15.6%	18.6%	0.08	17.8%	17.5%	−0.01
**DHHS region (%)**						
Region 1: CT, ME, MA, NH, RI, VT	4.5%	6.1%	0.07	5.6%	5.6%	0.00
Region 2: NJ, NY, PR, VI	8.0%	9.9%	0.07	9.2%	9.2%	0.00
Region 3: DE, DC, MD, PA, VA, WV	9.6%	9.0%	−0.02	9.2%	9.2%	0.00
Region 4: AL, FL, GA, KY, MS, NC, SC, TN	36.1%	22.1%	**−0.31**	26.6%	26.8%	0.01
Region 5: IL, IN, MI, MN, OH, WI	14.8%	15.0%	0.01	14.9%	14.9%	0.00
Region 6: AR, LA, NM, OK, TX	10.1%	17.5%	**0.21**	15.2%	15.0%	0.00
Region 7: IA, KS, MO, NE	1.9%	2.9%	0.07	2.6%	2.6%	0.00
Region 8: CO, MT, ND, SD, UT, WY	0.9%	1.3%	0.05	1.2%	1.2%	0.00
Region 9: AZ, CA, HI, NV, AS, FS, GU, PU	9.8%	11.2%	0.05	10.8%	10.7%	0.00
Region 10: AK, ID, OR, WA	4.3%	5.1%	0.04	4.8%	4.8%	0.00
**Payer type (%)**						
Cash	0.2%	0.2%	0.00	0.2%	0.2%	0.00
Medicaid	0.1%	0.1%	0.00	0.1%	0.1%	0.00
Medicare Part D	34.8%	32.7%	−0.04	33.1%	33.3%	0.01
Medicare	41.3%	37.5%	−0.08	38.5%	38.6%	0.00
Third party	23.7%	29.5%	**0.13**	28.2%	27.8%	−0.01

^1^ SMD (absolute) ≥ 0.1, bolded in the table, indicates significance. aIIV3 = adjuvanted trivalent influenza vaccine; SMD = standardized mean difference; DHHS = U.S. Department of Health & Human Services; HD-IIV3e = high-dose trivalent influenza vaccine. Note that as part of IPTW, a pseudo-population is created, composed of individuals in the pre-IPTW population weighted by the inverse of their probability of receiving the treatment that they received, given the baseline covariates. It is possible for the sample size for each cohort post-IPTW to change, but because stabilized IPTW weights are used, the total sample remains similar.

**Table 2:** Baseline Clinical Characteristics—Post-IPTW.

	Pre-IPTW			Post-IPTW		
Vaccine Cohort Characteristics	aIIV3 *n* = 798,255	HD-IIV3e *n* = 1,654,162	SMD ^1^	aIIV3 *n* = 798,987	HD-IIV3e *n* = 1,655,979	SMD ^1^
**Month of influenza vaccination (%)**						
August	10.7%	1.5%	**−0.39**	4.5%	4.6%	0.01
September	30.6%	29.4%	−0.03	29.6%	29.7%	0.00
October	41.1%	45.4%	0.09	43.9%	44.0%	0.00
November	12.0%	16.1%	**0.12**	14.8%	14.7%	0.00
December	4.0%	5.4%	0.07	5.0%	4.9%	0.00
January	1.7%	2.2%	0.04	2.1%	2.1%	0.00
**CCI score (%)**						
0	52.9%	52.1%	−0.02	52.3%	52.3%	0.00
1	21.2%	21.2%	0.00	21.3%	21.2%	0.00
2	13.0%	13.2%	0.01	13.2%	13.1%	0.00
3+	13.0%	13.5%	0.01	13.3%	13.3%	0.00
**Mean CCI score**	1.0	1.0	0.02	1.0	1.0	0.00
SD	1.4	1.4		1.4	1.4	
Median	0	0		0	0	
**Pre-index comorbidities (%)**						
Asthma	4.0%	3.9%	0.00	4.0%	3.9%	−0.01
Blood disorders	0.3%	0.3%	0.00	0.3%	0.3%	0.00
Chronic lung disease	9.2%	9.4%	0.01	9.3%	9.3%	0.00
Diabetes	22.3%	23.1%	0.02	22.6%	23.0%	0.01
Heart disease	14.0%	14.3%	0.01	14.3%	14.1%	−0.01
Kidney disorders	10.3%	10.5%	0.01	10.4%	10.4%	0.00
Liver disorders	2.6%	2.6%	0.00	2.6%	2.6%	−0.01
Neurological or neurodevelopmental conditions	5.3%	5.4%	0.01	5.4%	5.4%	0.00
Weakened immune system ^2^	11.3%	11.1%	−0.01	11.3%	11.1%	−0.01
IBD	0.7%	0.7%	−0.01	0.7%	0.7%	0.00
Composite of the above	50.7%	51.4%	0.01	51.2%	51.2%	0.00
**Indicators of frail health status (%)**						
Home oxygen use	5.2%	5.6%	0.02	5.5%	5.5%	0.00
Wheelchair use	2.8%	3.2%	0.02	3.0%	3.1%	0.01
Walker use	3.8%	3.9%	0.01	3.9%	3.9%	0.00
Dementia	1.5%	1.5%	0.00	1.6%	1.5%	0.00
Urinary catheter use	0.2%	0.2%	0.00	0.2%	0.2%	0.00
Falls	0.9%	0.9%	0.00	0.9%	0.8%	0.00
Fractures	0.5%	0.6%	0.00	0.6%	0.6%	0.00
Composite of the above	11.9%	12.5%	0.02	12.4%	12.3%	0.00
**Indicators of health-seeking behavior (%)**						
Cataracts	8.5%	8.2%	−0.01	8.4%	8.2%	−0.01
Eyelid disorders	1.3%	1.2%	−0.01	1.3%	1.2%	−0.01
Hemorrhoids	2.1%	2.0%	−0.01	2.0%	2.0%	0.00
Ingrown nail	1.0%	0.9%	−0.01	1.0%	0.9%	−0.01
Lipomas	0.3%	0.2%	0.00	0.3%	0.2%	0.00
UTI	6.6%	6.6%	0.00	6.6%	6.6%	0.00
Wound of hand or finger	0.5%	0.5%	0.00	0.5%	0.5%	0.00
Composite of the above	18.5%	18.0%	−0.01	18.3%	18.0%	−0.01
**Pre-index hospitalization (%)**	8.3%	8.2%	0.00	8.2%	8.2%	0.00
**Mean pre-index outpatient pharmacy costs**	USD 2109	USD 2154	0.02	USD 2111	USD 2158	0.02
SD	USD 6801	USD 6824		USD 6050	USD 6726	
Median	USD 530	USD 547		USD 527	USD 551	
**Mean inpatient costs**	USD 874	USD 857	0.00	USD 889	USD 853	0.00
SD	USD 8392	USD 8502		USD 8529	USD 8479	
Median	USD 0	USD 0		USD 0	USD 0	
**Mean outpatient medical costs (excluding ER)**	USD 3830	USD 3792	−0.02	USD 3861	USD 3759	−0.02
SD	USD 14,401	USD 14,194		USD 14,448	USD 14,147	
Median	USD 641	USD 619		USD 641	USD 615	
**Mean ER costs**	USD 193	USD 189	0.01	USD 195	USD 188	0.00
SD	USD 1059	USD 1046		USD 1071	USD 1043	
Median	USD 0	USD 0		USD 0	USD 0	
**Mean TOTAL pre-index costs ^3^**	USD 7006	USD 6991	0.00	USD 7056	USD 6959	0.00
SD	USD 19,559	USD 19,840		USD 19,733	USD 19,727	
Median	USD 2178	USD 2191		USD 2183	USD 2190	

^1^ SMD (absolute) ≥ 0.1, bolded in the table, indicates significance. ^2^ Including: HIV/AIDS; metastatic cancer and acute leukemia; lung or upper digestive or other severe cancer; lymphatic, head, neck, brain, or major cancer; breast, prostate, colorectal, or other cancer; and disorders of immunity. ^3^ TOTAL = outpatient pharmacy + inpatient + outpatient medical +ER. aIIV3 = adjuvanted trivalent influenza vaccine; CCI = Charlson Comorbidity Index Score; HD-IIV3e = high-dose trivalent influenza vaccine; IBD = Inflammatory bowel diseases (ulcerative colitis and Crohn’s disease); SD = standard deviation; SMD = Standardized mean difference; UTI = urinary tract infection.

**Table 3:** Economic Outcomes—Predicted Mean Annualized Costs Obtained using Recycled Predictions—Post IPTW and GLM Adjustment.

Predicted Mean Annualized Cost	aIIV3 *n* = 798,987		HD-IIV3e *n* = 1,655,979		Incremental Mean	*p*-Value
	Mean	95% CIs	Mean	95% CIs		
All-cause total	USD 13,196	USD 13,133–USD 13,260	USD 13,221	USD 13,176–USD 13,275	USD 25.24	0.2720
Influenza-related total	USD 21.64	USD 19.91–USD 23.36	USD 21.92	USD 20.79–USD 23.17	USD 0.28	0.4000
Influenza-related hospitalizations	USD 22.98	USD 19.32–USD 27.21	USD 22.04	USD 19.68–USD 24.53	−USD 0.94	0.3200
Influenza-related ER	USD 4.15	USD 3.73–USD 4.61	USD 4.44	USD 4.18–USD 4.74	USD 0.29	0.1280
Influenza-related office visit	USD 2.01	USD 1.80–USD 2.25	USD 1.92	USD 1.77–USD 2.09	−USD 0.09	0.2620
Influenza-related pharmacy	USD 2.75	USD 2.68–USD 2.81	USD 2.77	USD 2.73–USD 2.80	USD 0.02	0.3020

Influenza-related total costs = sum of influenza-related component costs (hospitalizations, ER, office visit, pharmacy).

## Data Availability

The original de-identified data used in this analysis were obtained from and are the property of IQVIA. IQVIA has restrictions prohibiting the authors from making the data set publicly available. Interested researchers may contact IQVIA to apply to gain access to the study’s data in the same way the authors obtained the data (see https://www.iqvia.com/contact/sf).

## Supplementary Materials

The following are available online at www.mdpi.com/article/10.3390/vaccines9101146/s1, Table S1. Adjusted rVE (95% CI)—post-IPTW and Poisson regression—aIIV3 vs. HD-IIV3e, Figure S1. Proportion of patients with influenza-related HCRU over the variable follow-up period (post-IPTW), Figure S2. Mean annualized influenza-related healthcare costs over the variable follow-up period (Post-IPTW), Table S2: All-cause HCRU and annualized cost during the post-index period-post-IPTW.
