# Supplementary material for: Comparing the Clinical and Economic Outcomes Associated with Adjuvanted versus High-Dose Trivalent Influenza Vaccine among Adults Aged ≥ 65 Years in the US during the 2019–20 Influenza Season—A Retrospective Cohort Analysis
Source: Vaccines (Basel). 2021 Oct 8;9(10):1146. doi: 10.3390/vaccines9101146 (PMC8540032; doi:10.3390/vaccines9101146)
Supplement: Supplementary file 1 [file vaccines-09-01146-s001.zip › vaccines-1369319-supplementary.pdf]

Supplementary Material

Table S1. Adjusted rVE (95% CI) – post-IPTW and Poisson regression – aIIV3 vs. HD-IIV3e

	Adjusted rVE			
**Measure**	**rVE**	**LCL**	**UCL**	**p-value**
**MAIN ANALYSIS**				
Influenza-related hospitalizations/ER visits	3.1%	-2.8%	8.6%	0.2978
All-cause hospitalizations	-0.7%	-1.6%	0.3%	0.1848
Any CRD-related hospitalization/ER visit	0.9%	0.0%	1.7%	0.0483
Pneumonia hospitalizations/ER visit	1.7%	-0.4%	3.7%	0.1153
Asthma/COPD/bronchial hospitalizations/ER visits	0.9%	-0.9%	2.6%	0.3308
Coronary artery hospitalizations/ER visits	-1.7%	-3.5%	0.1%	0.0625
MI hospitalizations/ER visits	-4.5%	-9.0%	-0.2%	0.0396
Congestive heart failure hospitalizations/ER visits	-0.1%	-2.0%	1.8%	0.9364
Cerebrovascular hospitalizations/ER visits	2.0%	-0.7%	4.5%	0.1393
Stroke hospitalizations/ER visits	2.0%	-0.7%	4.6%	0.1385
UTI hospitalizations	2.3%	-1.1%	5.6%	0.1846
**AGE GROUP 65 - 74**				
Influenza-related hospitalization/ER visit	4.5%	-4.0%	12.4%	0.2854
Any CRD-related hospitalizations/ER visits	1.4%	0.0%	2.7%	0.0435
**AGE GROUP 75 - 84**				
Influenza-related hospitalization/ER visit	6.6%	-3.2%	15.6%	0.1803
Any CRD-related hospitalizations/ER visits	0.6%	-0.8%	2.0%	0.3916
**AGE GROUP ≥85**				
Influenza-related hospitalization/ER visit	-8.8%	-25.0%	5.3%	0.2348
Any CRD-related hospitalizations/ER visits	-0.1%	-1.9%	1.7%	0.9568
**HIAP**				
Influenza-related hospitalization/ER visit	3.6%	-2.4%	9.3%	0.2359
Any CRD-related hospitalizations/ER visits	1.5%	0.5%	2.5%	0.0026
**SHORTENED INFLUENZA PERIOD**				
Influenza-related hospitalization/ER visit	2.8%	-4.2%	9.4%	0.4261
Any CRD-related hospitalizations/ER visits	0.8%	-0.2%	1.7%	0.1007

**DOUBLY ROBUST**				
Any CRD-related hospitalization/ER visit	3.0%	-2.9%	8.5%	0.3109
Influenza-related hospitalizations/ER visits	0.7%	-0.1%	1.6%	0.0942

aIIV3 = adjuvanted trivalent influenza vaccine; CRD = cardio-respiratory disease; ER = emergency room; HD-IIV3e = high-dose trivalent influenza vaccine; rVE = relative vaccine effectiveness; UTI = urinary tract infection

Figure S1. Proportion of patients with influenza-related HCRU over the variable follow-up period (post-IPTW)

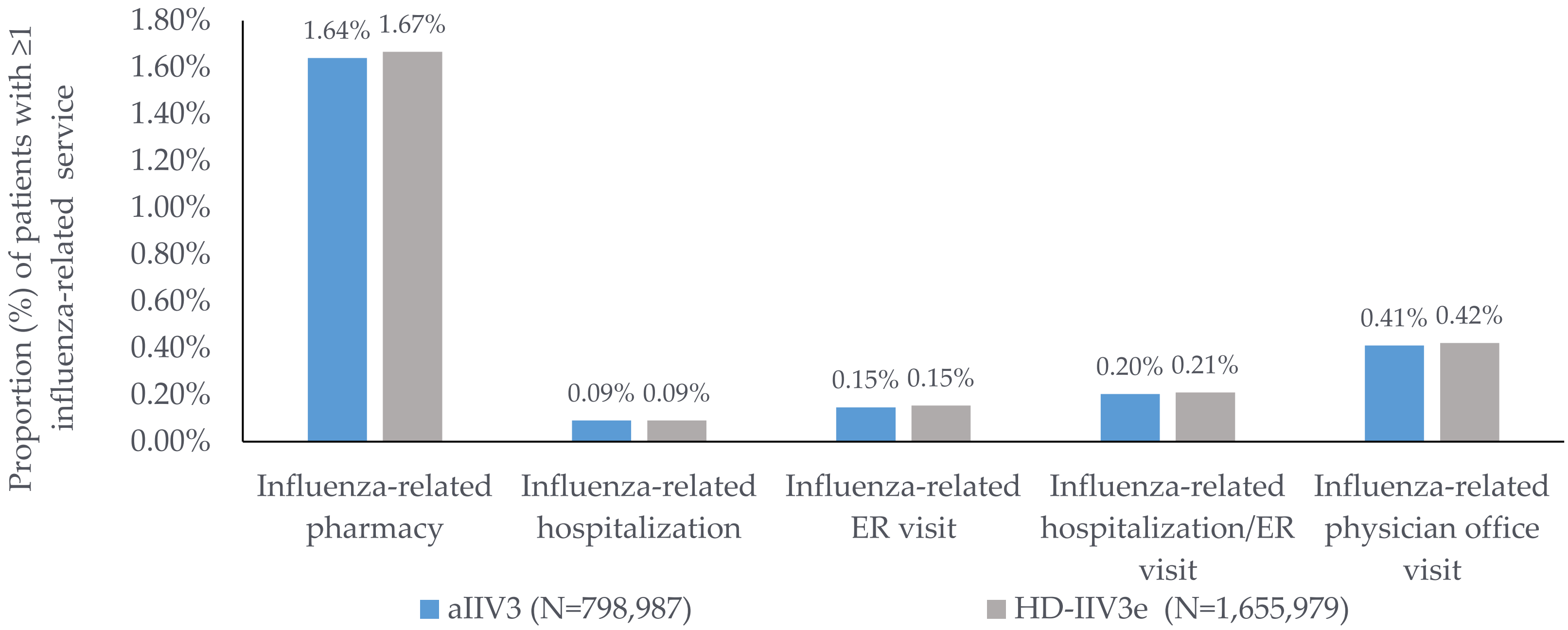

aIIV3 = adjuvanted trivalent influenza vaccine; ER = emergency room; HD-IIV3e = high-dose trivalent influenza vaccine

Figure S2. Mean annualized influenza-related healthcare costs over the variable follow-up period (Post-IPTW)

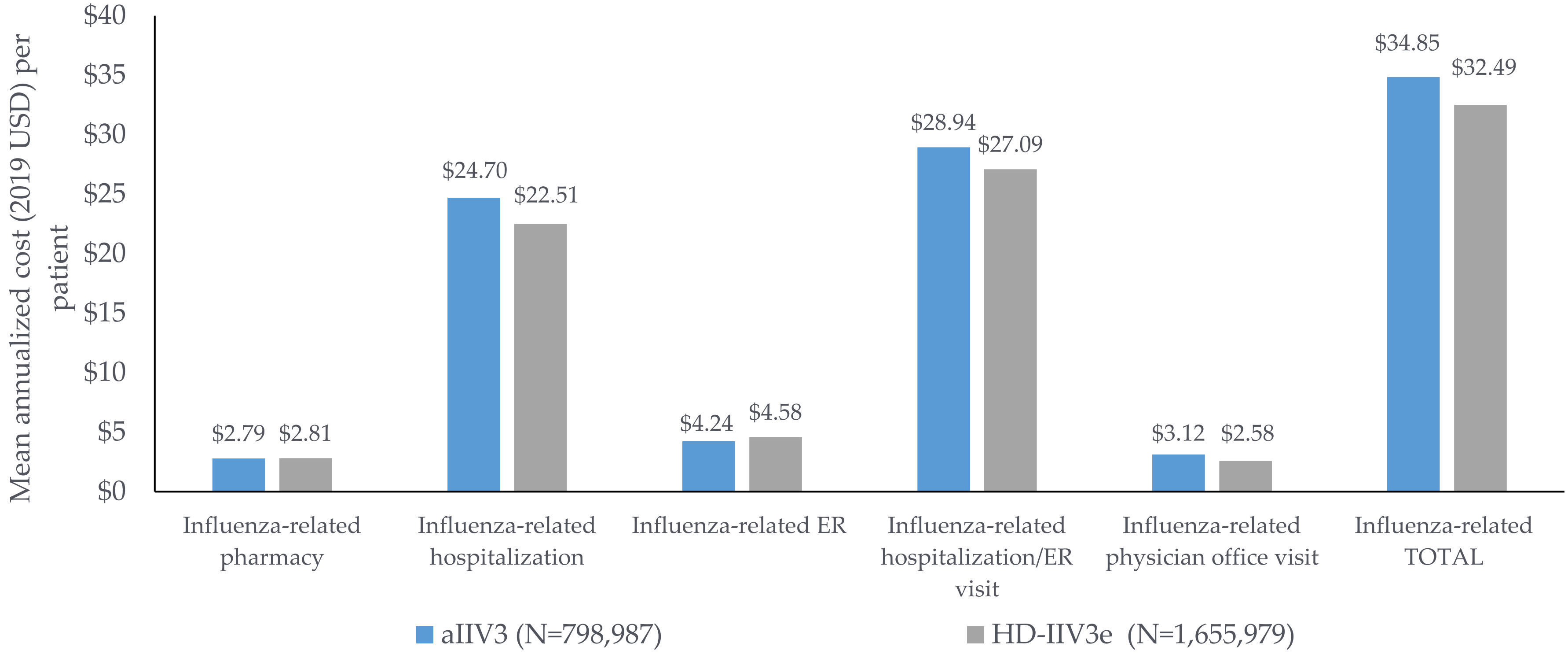

TOTAL = pharmacy + hospitalization + ER + outpatient office visit

Inpatient cost identified from CDM only

aIIV3 = adjuvanted trivalent influenza vaccine; ER = emergency room; HD-IIV3e = high-dose trivalent influenza vaccine

Table S2: All-cause HCRU and annualized cost during the post-index period - post-IPTW

Vaccine Cohort	aIIV3 (N=798,987)					HD-IIV3e (N=1,655,979)					
**Outcome (by visit type)**	**n**	**%**	**Mean**	**SD**	**Median**	**n**	**%**	**Mean**	**SD**	**Median**	**p-value[1]**
**Pharmacy**											
Patients with ≥1 pharmacy claim (n, %)	774,138	96.9%				1,611,321	97.3%				<.0001
Number of pharmacy claims per patient (mean, SD, median)			13.3	11.9	10			13.5	11.6	11	<.0001
**Inpatient hospitalization**											
Patients with ≥1 hospitalization (n, %)	16,034	2.0%				33,972	2.1%				0.0204
Number of hospitalizations per patient (mean, SD, median)			0.0	0.2	0			0.0	0.2	0	0.0796
ER											
Patients with ≥1 ER visit (n, %) (in either Dx or CDM based on service dates)	99,499	12.5%				207,433	12.5%				0.1045
Number of ER visits per patient (mean, SD, median)			0.2	0.6	0			0.2	0.6	0	0.3450
Physician office visits											
Patients with ≥1 physician office visit (n, %)	614,623	76.9%				1,264,841	76.4%				<.0001
Number of physician office visits per patient (mean, SD, median)			3.5	4.9	2			3.4	4.7	2	<.0001
**Total annualized Costs**											
Total annualized cost per patient	798,987	100%	$14,944	$64,666	$3,917	1,655,979	100%	$14,902	$49,619	$3,980	0.5730
Total annualized inpatient cost per patient	798,987	100%	$2,020	$25,805	$0	1,655,979	100%	$2,060	$25,402	$0	0.2486
Total annualized ER cost per patient	798,987	100%	$427	$2,629	$0	1,655,979	100%	$430	$2,614	$0	0.3992
Total annualized outpatient cost per patient	798,987	100%	$8,183	$53,942	$1,045	1,655,979	100%	$8,014	$34,334	$1,017	0.0030
Total annualized pharmacy cost per patient	798,987	100%	$4,313	$13,649	$1,028	1,655,979	100%	$4,397	$13,417	$1,083	<.0001

[1]Pair-wise comparisons conducted using weighted t-tests (mean) for continuous variables and weighted chi-square tests for categorical variables

Variable follow-up period: (index date + 14) to end of influenza season

aIIV3 = adjuvanted trivalent influenza vaccine; ER = emergency room; HD-IIV3e = high-dose trivalent influenza vaccine
